# Intramyocardial Injection of Pig Pluripotent Stem Cells Improves Left Ventricular Function and Perfusion: A Study in a Porcine Model of Acute Myocardial Infarction

**DOI:** 10.1371/journal.pone.0066688

**Published:** 2013-06-21

**Authors:** Xiaorong Li, Fengxiang Zhang, Guixian Song, Weijuan Gu, Minglong Chen, Bing Yang, Dianfu Li, Daowu Wang, Kejiang Cao

**Affiliations:** Department of Cardiology, the First Affiliated Hospital of Nanjing Medical University, Nanjing, Jiangsu Province, China; Clinica Universidad de Navarra, Spain

## Abstract

Induced pluripotent stem (iPS) cells have the potential to differentiate to various types of cardiovascular cells to repair an injured heart. The potential therapeutic benefits of iPS cell based treatment have been established in small-animal models of myocardial infarction (MI). We hypothesize that porcine iPS (piPS) cell transplantation may be an effective treatment for MI. After a 90-minute occlusion of the left anterior descending artery in a porcine model, undifferentiated piPS cells or PBS were injected into the ischemic myocardium. Cardiac function, myocardial perfusion and cell differentiation were investigated. One week after piPS cell delivery, global left ventricular ejection fraction (LVEF) significantly decreased in both the iPS group and the PBS group compared to the Sham group (*p*<0.05, respectively). Six weeks after piPS cell delivery, LVEF of the iPS group significantly improved compared to the PBS group (56.68% vs. 50.93%, *p* = 0.04) but was still lower than the Sham group. Likewise, the piPS cell transplantation improved the regional perfusion compared to the PBS injection (19.67% vs. 13.67%, *p* = 0.02). The infarct area was significantly smaller in the iPS group than the PBS group (12.04% vs. 15.98% *p* = 0.01). PiPS cells engrafted into the myocardium can differentiate into vessel cells, which result in increased formation of new vessels in the infarcted heart. Direct intramyocardial injection of piPS cells can decrease infarct size and improve left ventricular function and perfusion for an immunosuppressed porcine AMI model.

## Introduction

Novel embryonic stem cell (ESC)-like pluripotent stem cells, induced pluripotent stem (iPS) cells, were first generated from murines by transducing adult dermal fibroblasts with retroviruses encoding Oct3/4, Sox2, c-Myc, and Klf4 [1]. Subsequently, scientists have established different iPS cell lines from diverse species [2], [3], [4], [5]. In theory, iPS cells are capable of differentiating into various types of cells as needed, including cardiovascular cells [6], [7]. Due to limited plasticity of adult stem cells, mesenchymal stem cells (MSCs) and skeletal muscle cells have had slightly positive effects in clinical trials [8]. Compared to MSCs and ESCs, iPS cells are more accessible, and offer the possibility of generating patient-specific cell types for use in regenerative medicine. Therefore, iPS cells may have fewer ethical issues and eliminate the problem of immune rejection [9]. It may also represent the most promising candidate for personalized medicine. The generation of iPS cells offers the option of autologous transplantation, and provides a new tool for cellular therapy for ischemic heart disease.

The potential therapeutic benefits of iPS cell based treatment to improve the cardiac function have been established in small-animal models of myocardial infarction (MI) [10], [11], [12], [13], [14], [15], [16]. Canine and human iPS cells have been generated and transplanted to treat immunodeficient murine or porcine models of acute myocardial infarction (AMI) [17], [18], [19], but intramyocardial injection of porcine iPS (piPS) cells in a pig model of AMI has not yet been assessed. The pig, a common livestock, not only resembles the human heart in dimension, structure and function, but also preserves the immunological and physiological properties of human hearts [20]. A porcine AMI model can bridge the gap between treatment for small animals and treatment for humans [20]; therefore, they can serve as a powerful research tool for pre-clinical study and notably for transplantation medicine. Undoubtedly, ESCs are attractive cells that differentiate into different cardiovascular cells and improve an injured heart, but porcine ESCs have not been established to date [21], [22]. PiPS cells were successfully generated by three independent laboratories in 2009, which gave the best opportunity for pips transplantation [5], [23], [24]. Given these premises, we evaluate the feasibility and efficacy of piPS cell transplantation in the porcine AMI model.

## Methods

A detailed method is available in the online Text S1.

### Animal Care

All animals received humane care and the experimental procedures were performed in accordance with the “Guide for the Care and Use of Laboratory Animals” published by the US National Institutes of Health (NIH publication N0. 86/5-23, National Academy Press, Washington, DC, revised 1996). Study protocols were approved by the experimental animal care and ethical committee review board of the Nanjing Medical University, China.

### Experimental Protocol

Jiangsu Academy of Agricultural Sciences (Nanjing, P. R. China) provided all animals, including male and female pigs (weight 24.83±2.52 kg). After the initial single photon emission computed tomography (SPECT) scanning, all pigs were divided randomly into three groups: the Sham group (no LAD occlusion and without any intervention, n = 6), the phosphate buffered saline (PBS) group (received 2 ml of PBS after AMI creation, n = 9) and the iPS group (received 2 ml of undifferentiated enhanced green fluorescent protein (EGFP) labeled piPS cells after AMI establishment, n = 9). Undifferentiated EGFP-piPS cells (2×10^7^ in 2 ml) or the same volume of PBS solution were injected intramyocardially into infarct areas (8 sites) and peri-infarct areas (12 sites) under visualization by open-heart surgery one week after AMI model creation. SPECT and dual source computed tomography (DSCT) were performed to assess myocardial perfusion and cardiac function at the end of the 1st and 6th week after piPS cell or PBS delivery, respectively. All pigs were sacrificed at the end of the 7th week, and then heart tissues were harvested for macro and micro morphology study (Figure S1). To evaluate the safety of piPS cell transplantation, another two pigs were followed up to 4 months after piPS cell transplantation. MI animals received cyclosporine (10 mg/kg per day) after cell or PBS delivery.

### Cell Culture and Preparation

PiPS cell lines, kindly provided by Professor Lei Xiao (Shanghai Institutes for Biological Sciences, Chinese Academy of Sciences, shanghai, P. R. China), were generated from Danish Landrace pig primary ear fibroblasts by drug-inducible expression of defined factors including Oct4, Sox2, Klf4, c-Myc, Nanog and Lin28 [5]. The cell culture method is detailed in the online Text S1.

### AMI Model Creation and piPS Cell Transplantation

**Figure 1:**
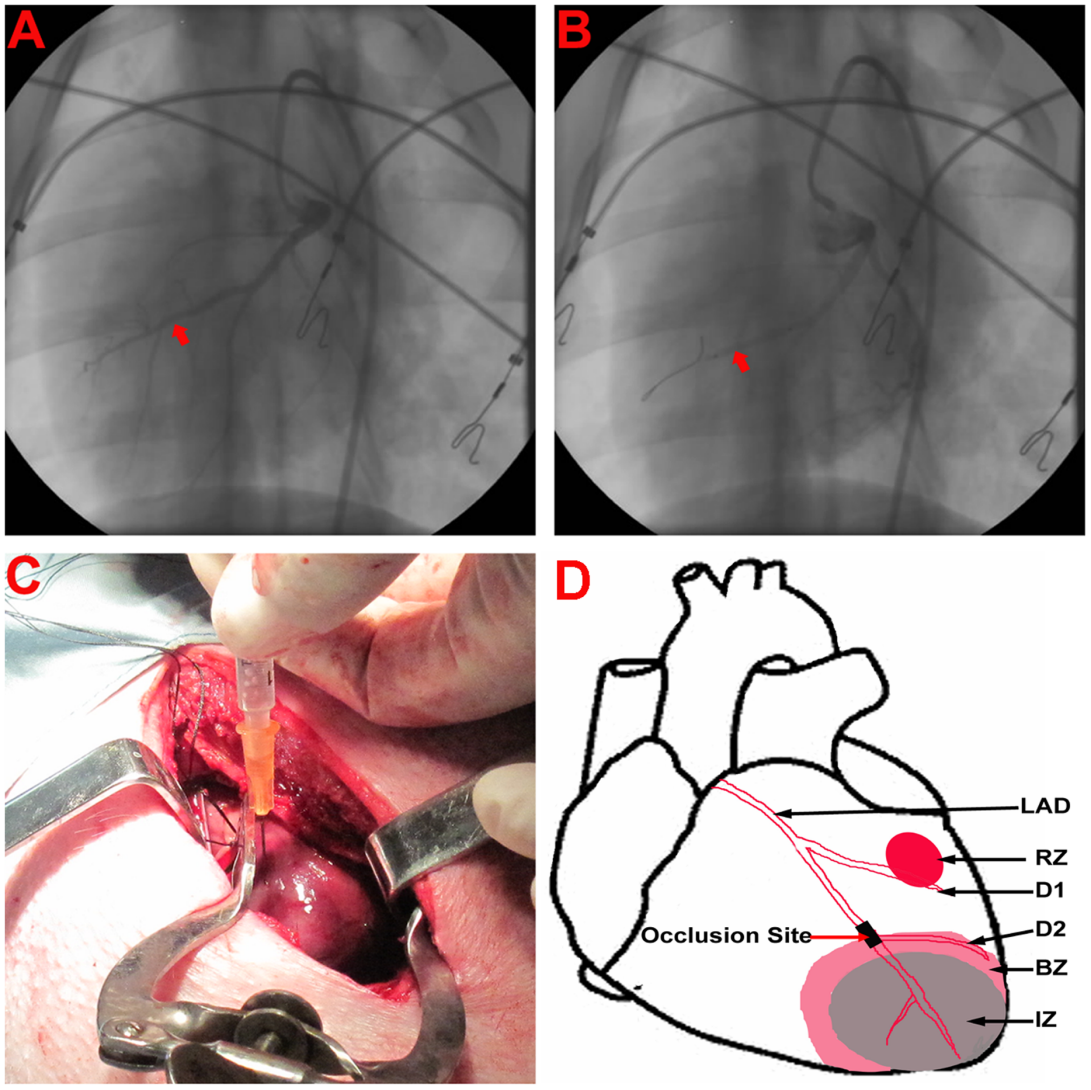
Creation of the AMI model. (**A**) The LAD indicated by the red arrow was completely normal and without any occlusion before inflation of the balloon. (**B**) Porcine AMI model was created by 90 min complete occlusion of the distal LAD by inflation of an over-the-wire balloon catheter (red arrow). (**C**) PiPS cell or PBS solution were injected directly into the IZ and BZ by open-heart surgery one week after AMI model creation. (**D**) The schematic diagram of the AMI heart. AMI = acute myocardial infarction; D1 = the first diagonal branch; D2 = the second diagonal branch; IZ = infarct zone; BZ = border zone; RZ = remote zone.

Under anesthesia, all pigs underwent a 90-minute occlusion of the left anterior descending artery (LAD) just distal to the first diagonal branch. To create AMI model, an over-the-wire balloon catheter was inflated (Figure 1A–B). One week later, pigs in the iPS group received 2×10^7^ piPS cells suspended in 2 ml of sterile PBS solution, which were injected both into the center of the infarct zone (IZ) for 8 sites and along the border zone (BZ) for 12 sites (0.1 ml per injection, 1×10^6^ cells per site) (Figure 1C–D). The same volume of PBS was injected into the corresponding sites of the PBS group. Sham-operated animals, in which the LAD was not occluded, were used as normal controls. AMI model creation and piPS cell transplantation are detailed in the online Text S1.

### Myocardial Perfusion and Cardiac Function Assessed by SPECT and DSCT

The methods have been previously described by our group [25] and are also detailed in the online Text S1.

### Analysis of Transplanted Cell Differentiation and Myocardial Vascular Density by Immunofluorescence (IF) and Immunohistochemical (IHC) Reactions

IF and IHC examination are detailed in the online Text S1.

### Gross Pathology, Histology and Ultrastructure Examination

Seven weeks after piPS cell delivery, the hearts were excised. Triphenyltetrazolium chloride (TTC) and hematoxylin & eosin (HE) staining were used to determine the infarct size and teratoma formation. Masson’s trichrome staining was performed to visualize the collagen content and transmission electron microscopy was performed to determine the ultrastructure alterations. All methods are detailed in the online Text S1.

### Statistical Analysis

Continuous variables were expressed as mean ± SD (standard deviation) and compared by *t*-test or one-way analysis of variance (ANOVA) test. Kruskal-Wallis H tests and Mann-Whitney U tests were used when the data did not met the normal distribution criteria or homogeneity of variance. Categorical variables were presented as a percentage ratio and compared by chi-square test unless otherwise indicated. For all the statistical analyses, *p*<0.05 was considered to be significant. *P* values are two-sided. Statistical analysis was performed using the SPSS software (Version 16.0, SPSS Inc., Chicago, Illinois).

## Results

### AMI Model Creation and Hemodynamic Analysis

During this study, 8 of 26 pigs died, including two during the initial SPECT examination. Six pigs could not tolerate the LAD occlusion and died from ventricular fibrillation (VF) within 90 minutes of the LAD occlusion even though defibrillation was performed. Therefore, the total procedural mortality rate was 30.77% in this study. No more pig deaths occurred once the piPS cells were injected into myocardium and the chest was then closed. For all animals, electrocardiography (ECG) showed that the ST segment in the V1–V3 leads was elevated and a pathological Q wave formed after 90 minutes of LAD occlusion. Some pigs died during this procedure because of ventricular tachycardia (VT) and VF. Left ventricular angiography was performed at baseline immediately after AMI for evaluation of wall motion (data not shown). The baseline characteristics and hemodynamic data at baseline and 7 weeks after cell delivery are presented in Table S1. There was no significant difference between sex, body weight, and breeding conditions among the three groups. Left ventricular end-diastolic pressure (LVEDP) was significantly higher in the PBS group at the 7th week, but there was no difference in other hemodynamic parameters, such as left ventricular end-diastolic pressure (LVSP), aortic systolic pressure (Ao-SP) and aortic diastolic pressure (Ao-DP), among the three groups. This result indicates that elevated LVEDP induced by MI can be attenuated by piPS cells treatment.

### Improvement of piPS Cell Engraftment on Myocardial Perfusion

**Figure 2:**
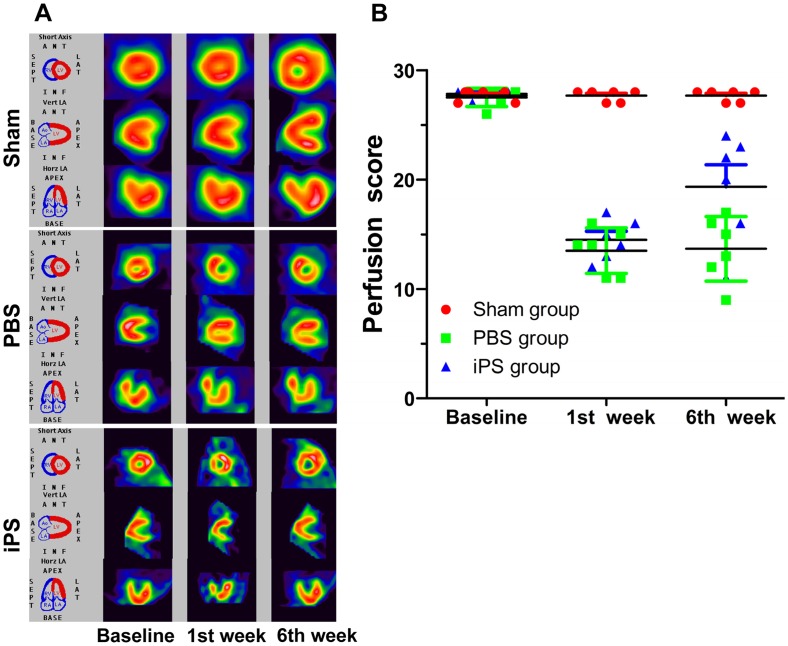
Myocardial perfusion determined by SPECT in three different groups. (**A**) SPECT demonstrated the myocardial perfusion of the iPS group significantly decreased one week after cell engraftment, but improved significantly from different axes six weeks later. (**B**) The perfusion scores of the three groups at different times revealed that piPS cell treatment improved the myocardial blood perfusion.

To evaluate piPS cell treatment efficacy, SPECT was performed to assess myocardial perfusion at baseline and first and sixth week after cell delivery for each animal. Figure 2A shows that cardiac perfusion at baseline were similar among the three groups. However, the first week after cell or PBS injection, the cardiac perfusion in both the iPS group and the PBS group were significantly reduced compared to baseline (Figure 2A–B). Six weeks later, the myocardial perfusion score of the iPS group was significantly improved compared to the PBS group (19.33±4.97 vs. 13.67±2.94, *p* = 0.04) despite still being lower than the Sham group (19.33±4.97 vs. 27.67±0.52, *p*<0.01) (Figure 2B). Overall, direct injection of piPS cells improved myocardial perfusion significantly in porcine model of AMI.

### Improvement of piPS Cell Engraftment on Left Ventricle Function

**Figure 3:**
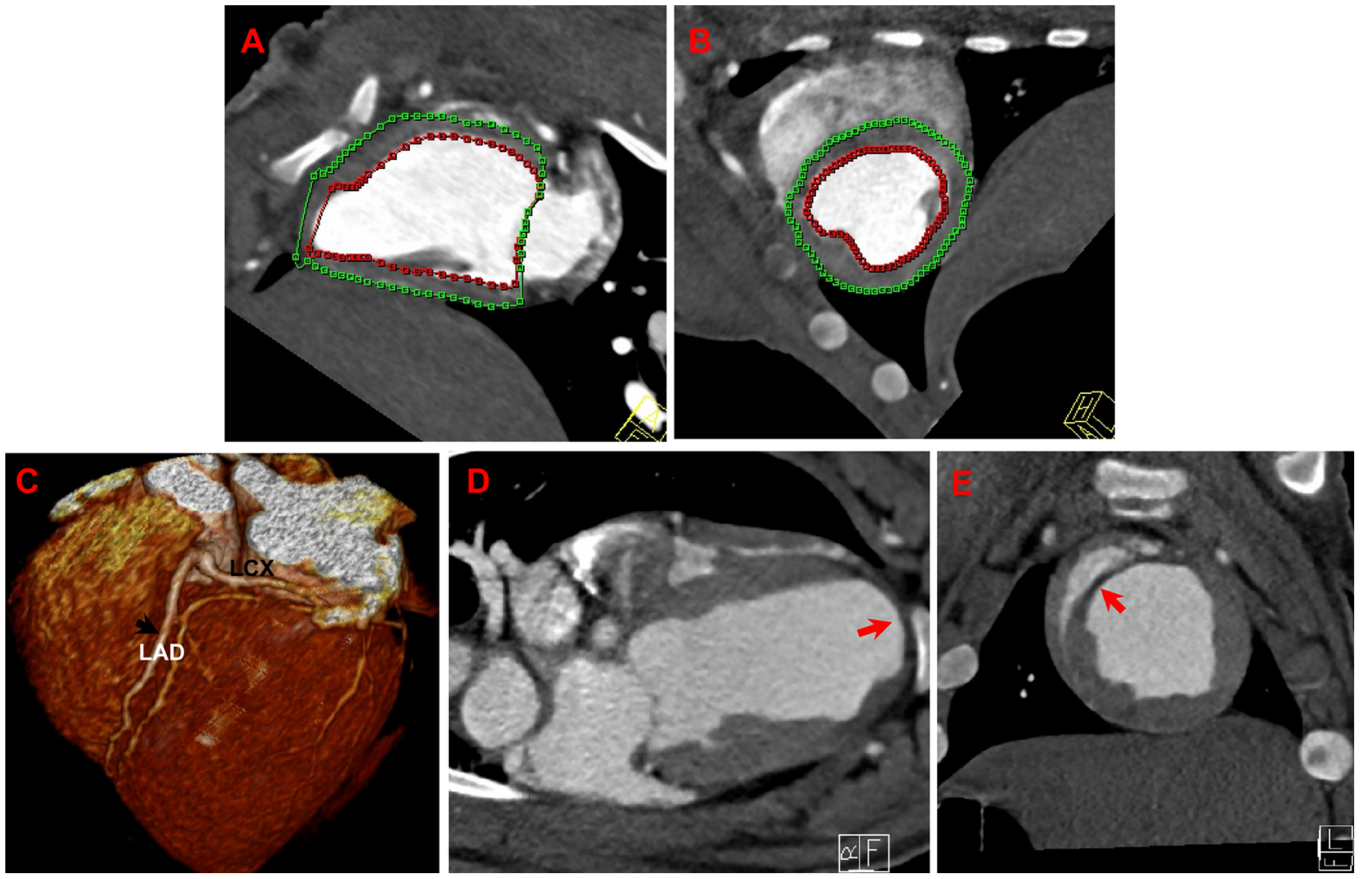
Assessment of global LV parameters and cardiac anatomy by DSCT. The tracing procedure for the left ventricle was automatically performed in the end-diastolic phase (**A**) and in the systolic phase (**B**) of the RR interval. A three-dimensional volume-rendered image of the LAD was constructed by DSCT. The LAD and LCX anatomy can be observed clearly (**C**). MI can be detected by DSCT in the PBS group and the iPS group both by long-axis and short-axis imaging (**D, E).** Enlarged left ventricle and aneurysms induced by MI can also be detected (**D).** The red arrow points to the site of MI, which was defined as a perfusion defect. MI = myocardial infarction.

**Figure 4:**
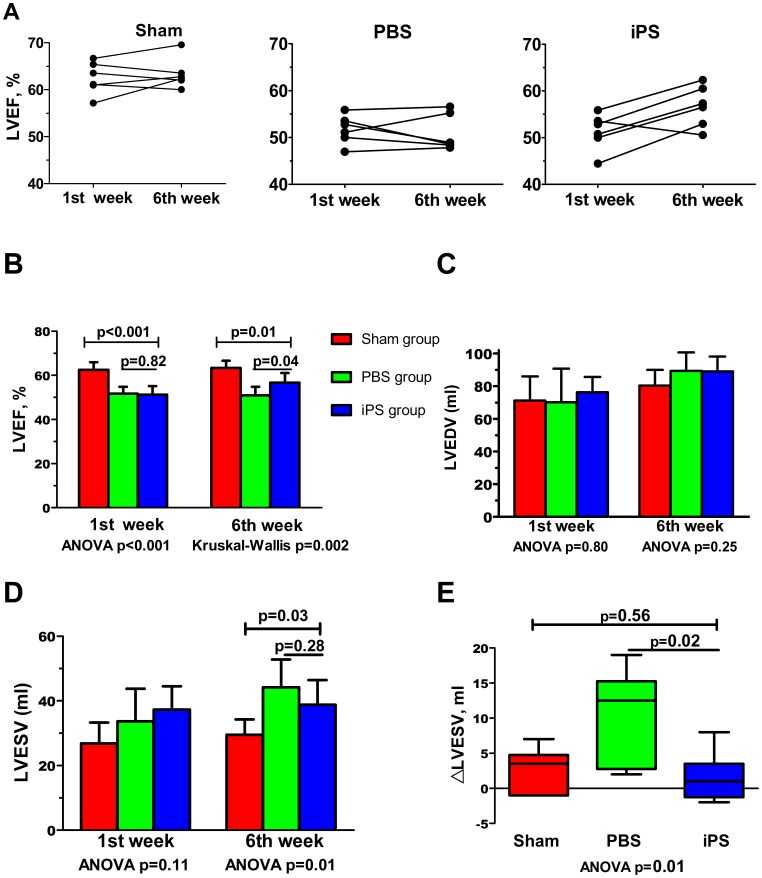
Intramyocardial transplantation of piPS cells improves cardiac function. (**A**) LVEF for each animal was shown on the 1st week and 6th week. The LVEF of the iPS group was equivalent to the PBS group but lower when compared to the Sham group on the 1st week. Six weeks later, LVEF of the iPS group significantly increased. (**B**) The LVEF on the 1st week did not differ between the iPS group and the PBS group (*p* = 0.82); however, LVEF of the iPS group significantly improved over the PBS group (*p* = 0.04) by the 6th week. (**C** and **D**) The LVEDV and LVESV were equivalent on the 1st week in three groups. Six weeks later, LVESV of the iPS group and PBS group increased (*p* = 0.01; however, there was no difference between the iPS and the PBS group (*p* = 0.28). (**E**) Demonstrated delta LVESV in the iPS group was significantly lower than the PBS group (*p* = 0.02). LVEF = left ventricular ejection fraction; LVEDV = left ventricular end-diastolic volume; LVESV = left ventricular end-systolic volume.

The DSCT examination was performed the first and sixth week after piPS cell or PBS injection. The tracing procedure for a smooth, concentric endocardial contour in the left ventricle, was automatically performed and adjusted manually if necessary (Figure 3A–B). First, the LAD anatomy and MI was confirmed (Figure 3C–E). Enlarged left ventricle and ventricular aneurysm induced by injured cardiac wall can be visualized by long-axis and short-axis imaging one week after piPS cell or PBS injection (Figure 3D and E). The global left ventricular ejection fraction (LVEF) and related parameters were then calculated. Parameters for LV function measured by DSCT were listed and summarized in Table S2. After the first week of piPS cell or PBS injection, LVEDV, LVESV and SV were qualitatively equivalent among the three groups. The LVEF of the PBS group and the iPS group were significantly decreased when compared to the Sham group (*p*<0.001), although was similar between the PBS group and the iPS group (51.72±3.09 vs. 51.25±3.94, *p* = 0.82) (Figure 4A–B). However, piPS cell treatment resulted in a significant improvement in LVEF of the iPS group compared to the PBS group (56.68±4.44 vs. 50.93±3.91, *p* = 0.04) six weeks after piPS cell transplantation, though this was still lower than the Sham group (56.68±4.44 vs. 63.37±3.26, *p* = 0.01). Thus, compared to the PBS injection, piPS cell intervention improved cardiac function after AMI, mainly due to the change of the LVESV but not LVEDV (Figure 4C–E).

### The Fate of Engrafted piPS Cells and Vessel Formation

**Figure 5:**
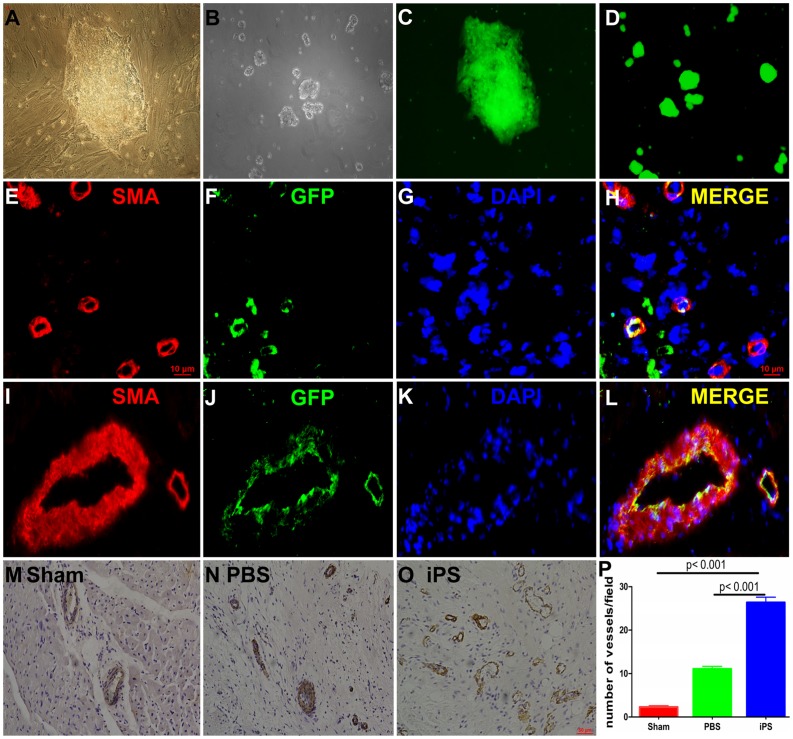
PiPS cell culture and the fate of engrafted cells. (**A**) PiPS cells were cultured on irradiated MEFs (200×). (**B**) PiPS cells were cultured on CM without feeder cells before transplantation. (**C** and **D**) PiPS cells displayed green fluorescence under fluorescence microscopy (100×). Frozen samples were stained with α-smooth muscle actin (α-SMA) and GFP. Nuclei were stained by DAPI. Histological analysis showed that piPS cells could differentiate into vessel cells in the IZ (**E–H,** 400×) and the BZ (**I–L**, 200×). (**E–L**) Red represents SMA, green represents GFP, and blue represents nuclear. H is the merge of E-G, L is the merge of the I-K. The representative neovascularization sections were stained with α-SMA of the (M) Sham group, (**N**) the IZ of the PBS group and (**O**) the IZ of the iPS group (200×). Vascular density (α-SMA staining) in the three groups seven weeks after cell delivery (**P**).

The EGFP-labeled piPS cells were cultured on mouse embryonic fibroblasts (MEF) cells and demonstrated ESC morphology (Figure 5A) and other appropriate stem cell markers of pig ES cells as described by Wu et al. [5]. Before transplantation, piPS cells were cultured on condition medium (CM) for 3–5 days without feeder cells (Figure 5B). The green fluorescence of the piPS cells can be detected under fluorescence microscopy (Figure 5C, D). As observed in human ESCs, the piPS cells stained positive for ES cell surface markers, including Nanog, SSEA-3, and TRA-1-81 (Figure S2). To test pluripotency in vivo, piPS cells were injected intramuscularly into nude mice. We observed the teratomas contained various tissues of three germ layers, including endoderm, mesoderm and ectoderm four weeks after piPS cell injection (Figure S3).

Seven weeks after piPS cell transplantation, myocardium tissue from the iPS group was examined by IF. In the IZ (Figure 5E–H) and BZ (Figure 5I–L) of the iPS group, double anti-α smooth muscle cell actin (α-SMA)/GFP cells were observed via co-staining of α-SMA and GFP, which indicated the piPS cells differentiated into SMA positive myocardial vessel cells. Seven weeks after cell transplantation, IHC staining for SMA antibodies indicated that there was significant angiogenesis in the iPS group with more SMA-positive capillaries present in the IZ compared to the other two groups (Figure 5M–O). Quantitative evaluation of the number of SMA-positive per field (×200) indicated that vascular density was much higher in the IZ of the iPS group than that of the PBS treated group and the Sham group (PBS group vs. iPS group, 11.09±3.93 vs. 26.38±8.17, *p*<0.001; Sham group vs. iPS group, 2.31±2.08 vs. 26.38±8.17, *p*<0.001, Figure 5P).

**Figure 6:**
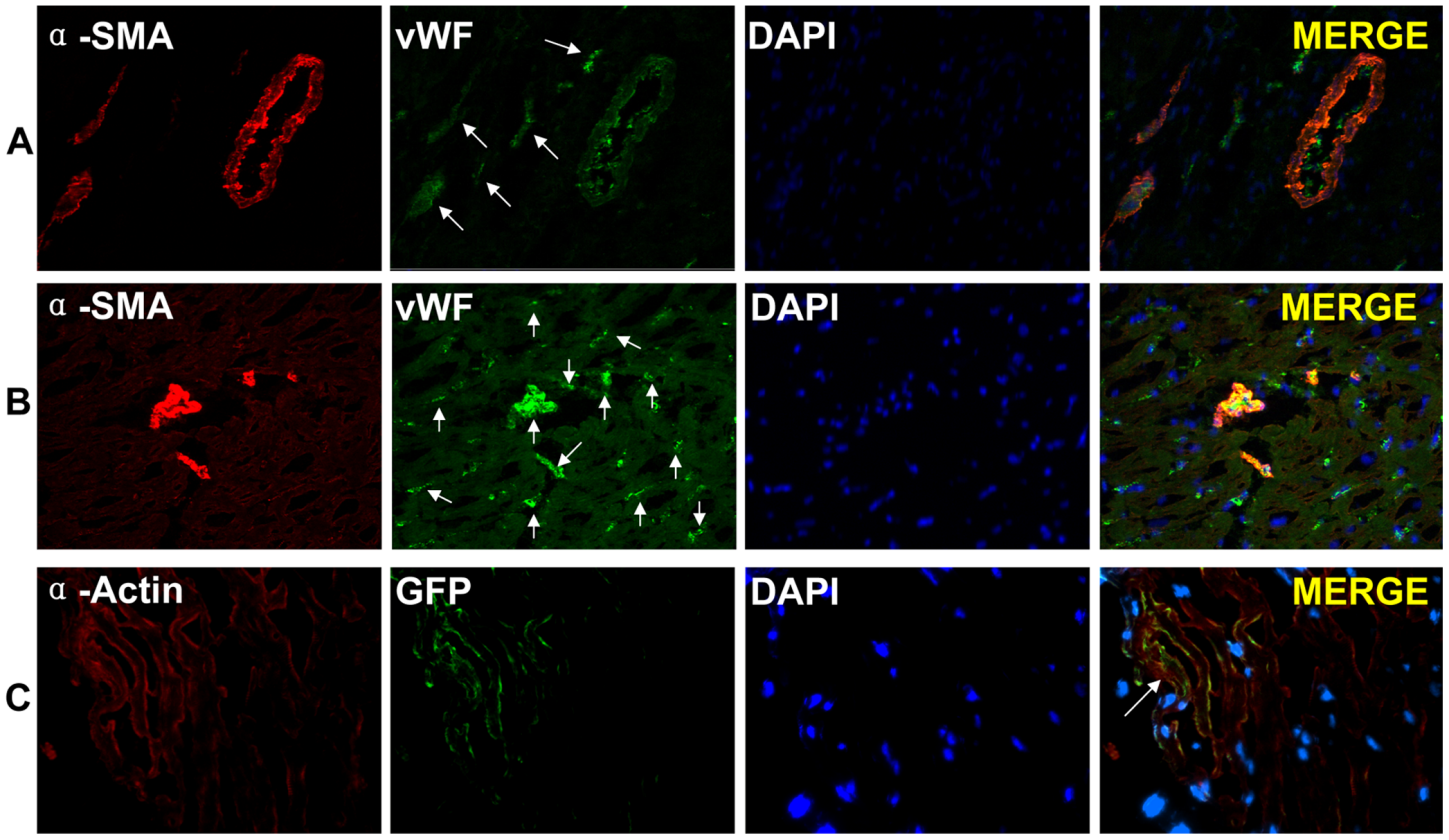
Immunostaining for cardiac and vascular endothelial cell markers. There were significantly more Willebrand factor (vWF) positive cells in the BZ of the iPS group (**B**) than of the PBS group per field (×200) (**A**). Immunoﬂuorescence staining showed piPS cells tagged with green ﬂuorescent protein (GFP) differentiated into cardiomyocytes seven weeks after engraftment (×400) (**C**).

To determine the formation of vascular endothelium and vessel maturation, co-staining of α-SMA and von Willebrand factor (vWF) was observed under confocal microscopy (Figure 6A–B). The number of vWF-positive cells in the BZ of the iPS group was significantly greater than that of the PBS group per field (×200) (PBS group vs. iPS group, 14.63±4.21 vs. 34.89±10.54, *p*<0.001). Figure 6C shows only a few clusters of GFP-positive piPS cells can penetrate into the BZ and differentiate into cardiomyocytes. These results demonstrate that piPS cells can survive in vivo as well as promote arteriogenesis and angiomyogenesis.

### Histopathology Findings

**Figure 7:**
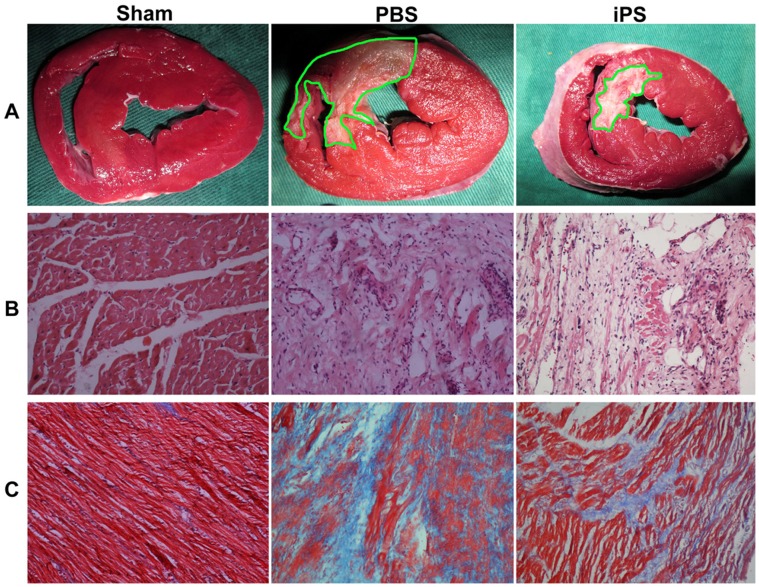
Representative morphological photomicrographs of histopathology examination. (**A**) Short-axis TTC staining showed that the percentage of the infarction area was smaller in the iPS group than in the PBS group. Infarct tissue was shown in white and demarcated by green lines. (**B**) Note the HE examination (200×) for the LV of the Sham group (left), border zone of the PBS group (middle) and the iPS group (right). (**C**) PiPS cell delivery reduces myocardial fibrosis. Note the Masson’s Trichrome staining (200×) of the LV of the Sham group (left), border zone of the PBS group (middle) and the iPS group (right). Viable myocardium was stained bright red and fibrosis was blue.

Figure 7A shows that the infarction area of the iPS group was statistically smaller than that of the PBS group seven weeks following the piPS cell injection (15.98±1.57% vs. 12.04±1.46%, *p* = 0.01). HE staining demonstrated normal histopathological features without any sign of necrosis and fibrosis in the Sham group; however, cardiomyocyte necrosis and neutrophil accumulation within interstitial spaces were disclosed in the iPS and PBS group on the seventh week. Nevertheless, more myocardium islets were detected in the BZ of the iPS group compared to the PBS group (Figure 7B). Masson trichrome staining showed a profound decrease in collagen in the BZ of the left ventricle wall in the iPS group compared to the PBS group (18±4% vs. 35±6%, *p*<0.001, Figure 7C).

### The Safety of Porcine piPS Cell Transplantation

**Figure 8:**
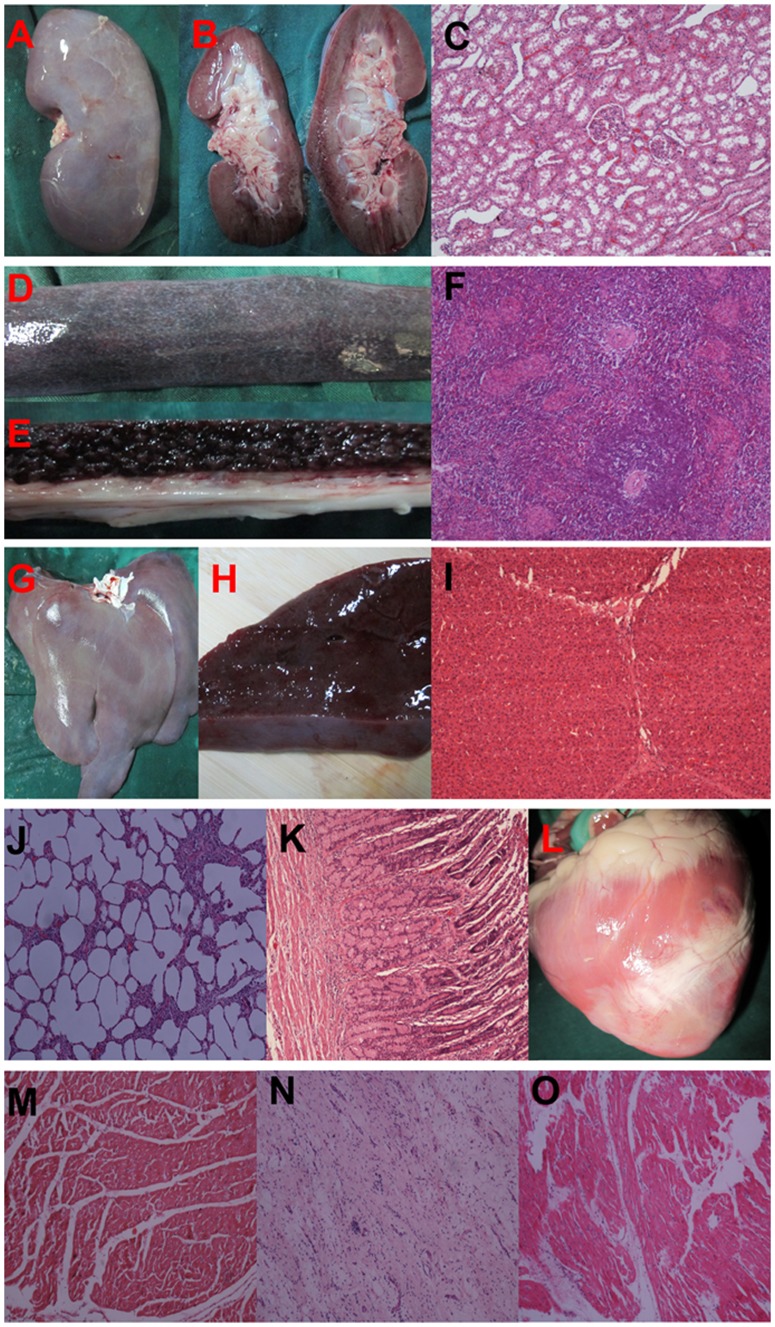
Lack of evidence for tumor formation in any organs in the iPS group. Sections stained with HE showed no signs of tumor formation upon histological examination in any animals not only in the gross appearance of the organ but also under the microscope. (**A–C**) Kidney; (**D–F**) Pancreas; (G–I) Liver; (**J**) Lung; (**K**) Stomach; (**L–O**) Heart; (**M**) HE of remote zone; (**N**) HE of infarct zone; (**O**) HE of border zone.

In all pigs, there were no tumors detected in any of the organs (heart, lung, liver, spleen or kidney) after inspection by naked eye seven weeks after intramyocardial injection of piPS cells. HE histology examination also did not find any signs of tumorigenesis (Figure 8). To evaluate the long-term safety of piPS cell transplantation, two additional pigs were followed up to 4 months after piPS cell transplantation. No tumor was found in any of the major organs in both pigs.

### Ultrastructure Changes of the Myocardium

The myocardium from the Sham group had a normal appearance with the nucleus in the center, normal mitochondria, a clear Z-line and sarcomere as well as no disruption in mitochondria or sarcomeres (Figure S4A). In contrast, the myocardium taken from the IZ of the PBS group were severely damaged (Figure S4B). Most of myocardium, including nucleus, mitochondria, clear Z-line and sarcomere were lost. Organelles and myofilaments were dramatically reduced, and at the same time, collagen fiber was found in the IZ to replace the cardiomyocytes. Myocardium taken from the RZ of the iPS group displayed the same morphological features as a healthy myocardium (Figure S4C). The myocardium from the BZ of the iPS group was slightly damaged (Figure S4D), including swollen mitochondria, small vacuoles formed in mitochondria and minor disorganization of the myofibrillar proteins compared to a healthy myocardium. The myocardium from the IZ of the iPS group was mainly composed by collagen fibers (Figure S4E and S4F), but the scarred areas were smaller compared to the PBS group. Therefore, in contrast to ineffective PBS treatment, targeted delivery of piPS cells preserved the ultrastructural characteristics in post ischemic myocardium; moreover, piPS cells did not generate cardiac tumorgenisis under electron microscopy.

## Discussion

### Main Findings

This study demonstrates that transplantation of piPS cells can improve cardiac perfusion and function in a clinically relevant AMI model, which may be associated with a significant increase of neovascularization.

### Engraftment of piPS Cells can Improve Ventricular Perfusion and Cardiac Function

The pig has long been used as a model for cellular replacement therapies. Since somatic stem cells cannot effectively differentiate into cardiac cells, ESCs are appealing for their totipotency. Porcine ESCs have yet to be established due to the poor understanding about pre-implantation development and culture conditions in vitro [26]. Initially, various publications reported the successful derivation of piPS cells [5], [23], [24] and several studies also showed that pig somatic cells could be reprogrammed into piPS cells [26], [27]. PiPS cell therapy has potentially provided a new option for the study of cellular translational medicine. In our current study, we first tested the intracoronary administration of piPS cells, and found there was no effect on the infarct area and on cardiac perfusion. The mechanism(s) is unknown and may be related to the first pass uptake phenomenon. Therefore, we chose a direct intramyocardial injection route to deliver the piPS cells to the target zones. Many studies have applied SPECT and DSCT to evaluate cardiac function and perfusion in the porcine model of AMI [25], [28], [29], [30]. Our results demonstrated that engraftment of piPS cells can lead to improvement in ventricular perfusion and cardiac function in a porcine AMI model using SPECT and DSCT examination. The LVEF in the iPS group improved significantly after the 6-week follow-up, which may have resulted from the LVESV changing. We also identified a significant reduction of infarct size by TTC staining after piPS cell injection. These results were in accordance with previous reports in small animal models [10], [11], [16]. Blin et al. [31] transplanted rhesus ESC-derived progenitors into the infracted myocardium of immunosuppressed nonhuman primates and their study did not show changes in global cardiac function and perfusion.

### Mechanisms of iPS Cells Engraftment on Cardiac Function

Until now, there were several possible mechanisms that are widely accepted regarding cell engraftment improving myocardial perfusion and cardiac function. The first mechanism involves cardiomyocytes differentiated from engrafted cells that directly repair the infarcted cardiomyocyte, thus improving cardiac function. The second involves neovascularization induced by new vessel cells differentiated from engrafted cells. Thirdly, paracrine factors such as vascular endothelial growth factor (VEGF), insulin-like growth factor, hepatocyte growth factor, and fibroblast growth factor released after engraftment may activate the endogenous cardiac progenitors to protect and repair the lost myocardium. In this study, we demonstrated that piPS cells transplanted into the infarcted myocardium in a porcine model could survive and differentiate into vessels cells and myocytes. We also found that significant neovascularization occurred in the IZ of the piPS cell treated group compared to the untreated hearts (Figure 5M–P). This mechanism has also been demonstrated by bone marrow-derived multipotent progenitor cells for AMI therapy [32] and iPS cell therapy for the AMI model in the small animal model [12]. These results suggest that improved ventricular function and cardiac perfusion may result in part from neovascularization.

Several studies have suggested that iPS cells have the ability to differentiate into diverse cardiovascular cells [7], [33]. Therefore, the present study was carried out to examine whether piPS cells can be used to prevent LV dysfunction and post infarction left ventricular remodeling in hearts suffering from AMI. Consequently, our data demonstrates that these donor cells engrafted in the targeted region can differentiate to new vessel cells and promote the myocardial neovascularization of the infarcted cardiomyocytes. In turn, this improves LV chamber function thus producing a beneficial effect. We did not observe obvious myocyte generation; this may due to the amount of myocardial injury is typically small in this model.

### The Safety of iPS Cell Therapy for Cardiac Disease

iPS cell technology has recently reached a milestone and received much attention by researchers and clinicians because of its potential applications in medical fields. However, many issues must be addressed regarding its clinical application including potential teratoma formation. Recently, our group reported that autologous iPS cell transplantation in rat hearts can induce in situ tumorigenesis at 2, 4 or 6 weeks. There is no current explanation for these findings. We hypothesize the tumors are caused by iPS cells that leake from the beating heart [34]. At the same time, Ahmed et al. [35] also reported that mice transplanted with undifferentiated iPS cells, in a model with permanent coronary artery ligation, were found to have intramural teratomas four weeks after the administration of iPS cells. Contrarily, in our study, there are no visible tumor-like structures in the heart during the six-week or four month follow-up after iPS cell transplantation. The following reasons are possible explanations for this occurrence. First, the observation time was not long enough and macro examination cannot find an early stage tumor. Second, the original cells used to induce the iPS cells are different from the cells Ahmed’s group used. Third, different animals have different reactions to iPS cell implantation. Nevertheless, our results were in accordance with several previous studies, which also transplanted iPS cells into an infarct heart [10], [11], [12]. Despite no tumor formation in this study, there is the need for a more in depth study of the translation of iPS technology into clinical use due to the potential long-term risk of tumor formation.

### Limitation

The present study has some limitations. First, we did not differentiate the piPS cells into cardiomyocte and cardiac progenitor cells; therefore, we cannot compare the effect of undifferentiated and differentiated cells on cardiac perfusion and function. This was partly because we wanted to let the piPS cells fully display its pluripotent ability to differentiate into cardiomyocte, smooth vascular cells, fibroblast and other conductive system cells in the heart. Secondly, the chronic myocardial ischemia model was not applied due to the large expenditure and long time frame. Another limitation involved not applying the intracoronary cell engraftment routine. This was because we did not find any improvement of LVEF and infarct size in our preliminary experiment. The last limitation occurred because we did not compare different doses and different types of stem cells and their effect on AMI. Future studies are needed to address these issues.

### Conclusion

In conclusion, the present study suggests that direct delivery of piPS cells could improve cardiac function and perfusion as measured by DSCT and SPECT in a porcine AMI model. The therapeutic effects may be due to increased revascularization and myogenesis. PiPS cells may have the potential to be used in ischemic cardiac diseases. Further studies are needed to explore the mechanism and long-term safety of this novel cell therapy.

## Supporting Information

Figure S1 The flow chart of the study. LAD = left anterior descending coronary artery; SPECT = Single photon emission computed tomography; DSCT = Dual-source computed tomography.
(TIF)

Figure S2 Pluripotency characteristics of piPS cell lines. PiPS colonies express Nanog (Left), SSEA3 (Middle), and Tra-1-81(Right). Scale bars: 50 um. piPS = porcine iPS.
(TIF)

Figure S3 Teratoma formation in immunodeficient mice injected with piPS cells demonstrate differentiation into the three germ layers. Teratoma is composed of various types of tissues: endoderm (Left), mesoderm (Middle), ectoderm (Right). Scale bars: 50 um.
(TIF)

Figure S4 Ultrastructure of the myocardium in different groups. (**A**) Healthy myocardium from the Sham group. (**B**) Myocardium taken from the infarct zone of the PBS group showed a large damaged area. (**C**) Myocardium taken from the remote zone of the iPS group. (**D**) Myocardium taken from the border zone of the iPS group was only slightly damaged. (**E**) Myocardium taken from the infarct zone of the iPS group was mainly composed of fibroblasts, and newly formed myocardium also appeared (showed by red squared portion). (**F**) A highly magnification photograph of the squared portion of **E**. No tumor signs were seen.
(TIF)

Table S1 The characteristics and hemodynamic data at the end of the 7th week after piPS cell transplantation.
(DOC)

Table S2 LV function parameters at the end of 1st and 6th week after piPS cell transplantation.
(DOC)

Text S1 Supporting Materials and Methods.
(DOC)
