# Supplementary figures and images for: Intramyocardial Injection of Pig Pluripotent Stem Cells Improves Left Ventricular Function and Perfusion: A Study in a Porcine Model of Acute Myocardial Infarction
Source: PLoS One. 2013 Jun 21;8(6):e66688. doi: 10.1371/journal.pone.0066688 (PMC3689724; doi:10.1371/journal.pone.0066688)

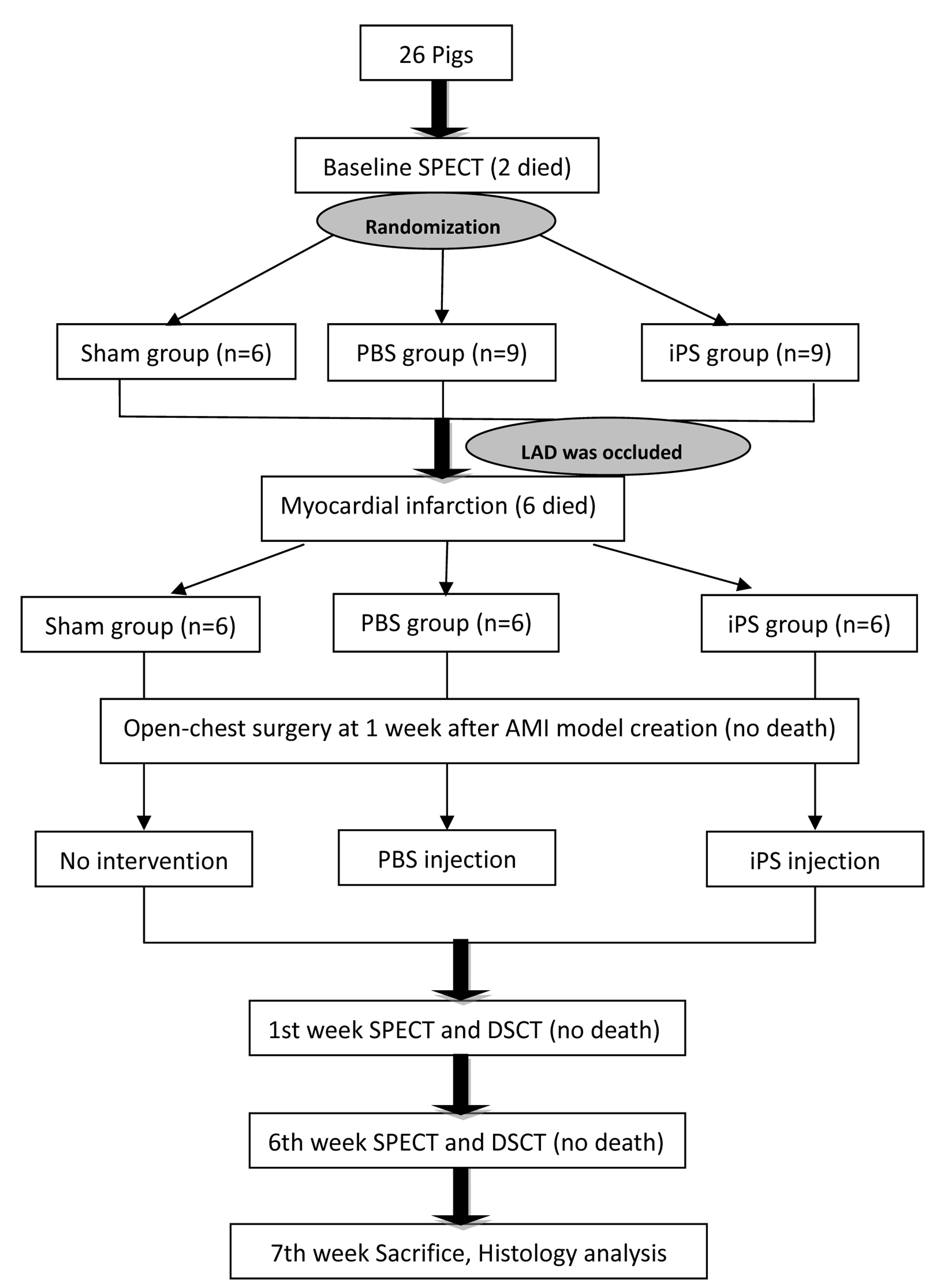

Supplement: Figure S1 — The flow chart of the study. LAD = left anterior descending coronary artery; SPECT = Single photon emission computed tomography; DSCT = Dual-source computed tomography. (TIF) [file pone.0066688.s001.tif]

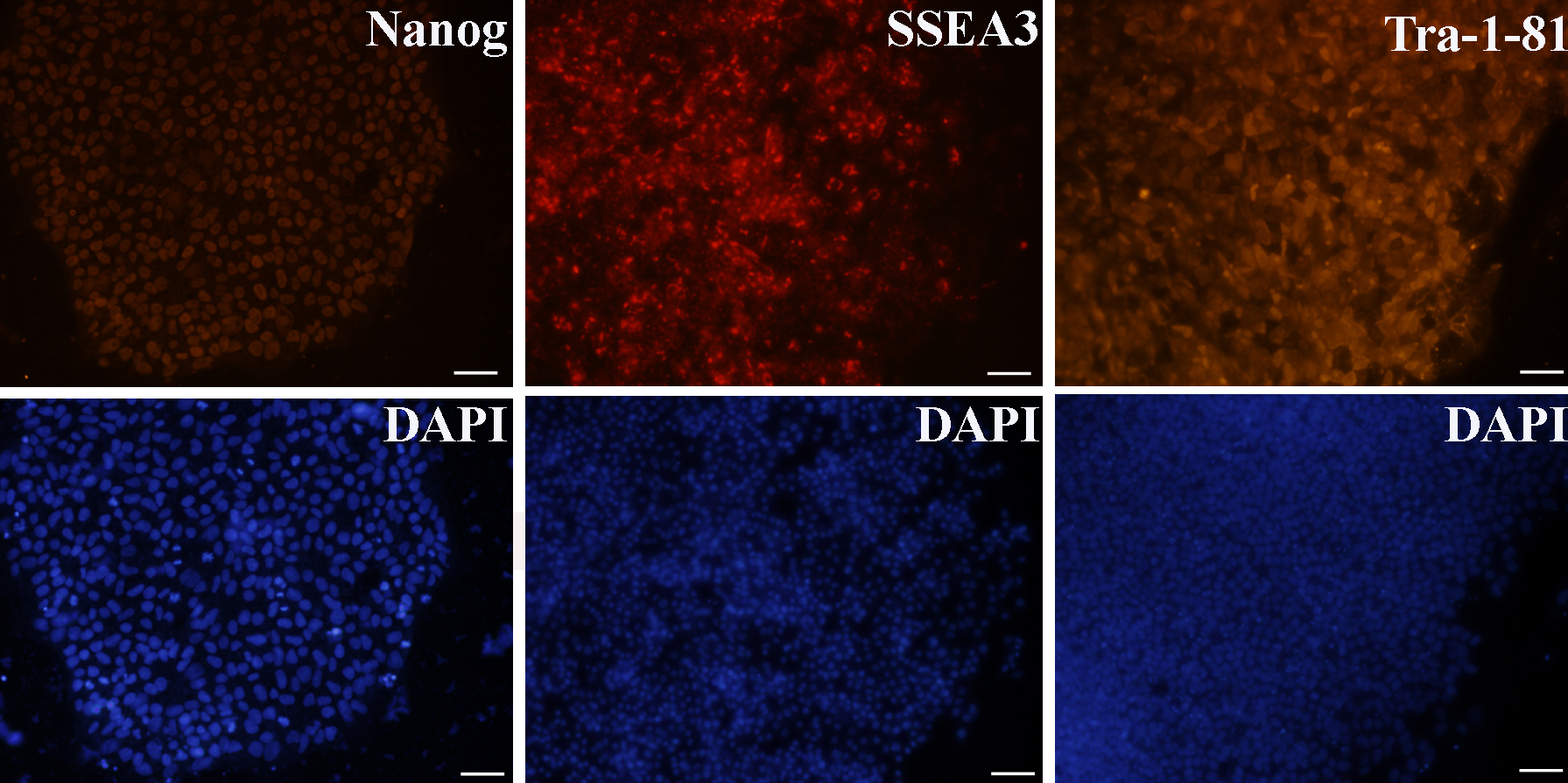

Supplement: Figure S2 — Pluripotency characteristics of piPS cell lines. PiPS colonies express Nanog (Left), SSEA3 (Middle), and Tra-1-81(Right). Scale bars: 50 um. piPS = porcine iPS. (TIF) [file pone.0066688.s002.tif]

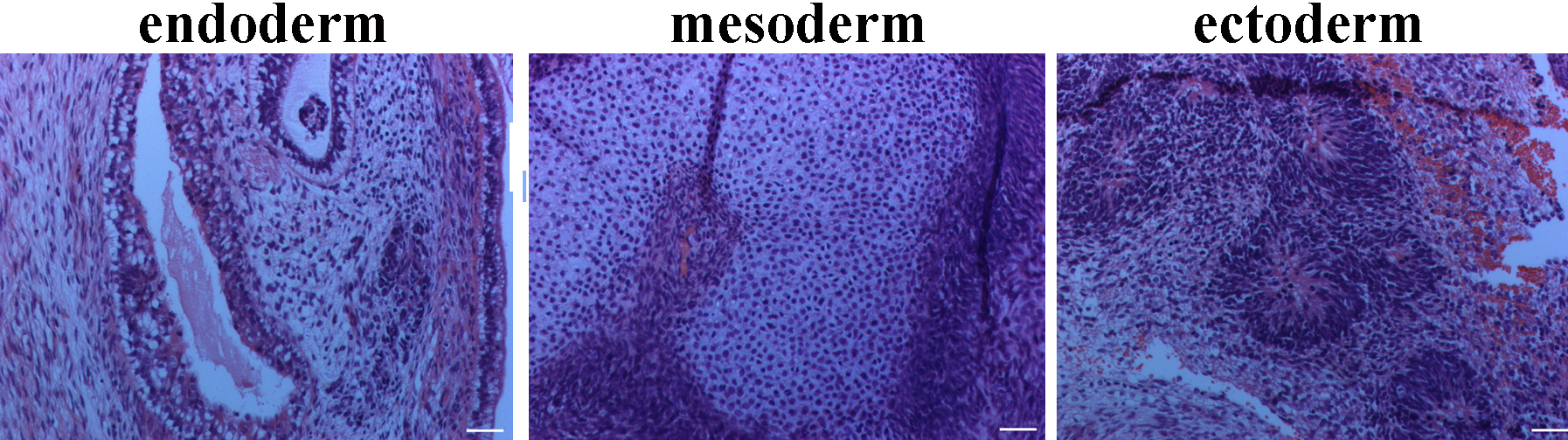

Supplement: Figure S3 — Teratoma formation in immunodeficient mice injected with piPS cells demonstrate differentiation into the three germ layers. Teratoma is composed of various types of tissues: endoderm (Left), mesoderm (Middle), ectoderm (Right). Scale bars: 50 um. (TIF) [file pone.0066688.s003.tif]

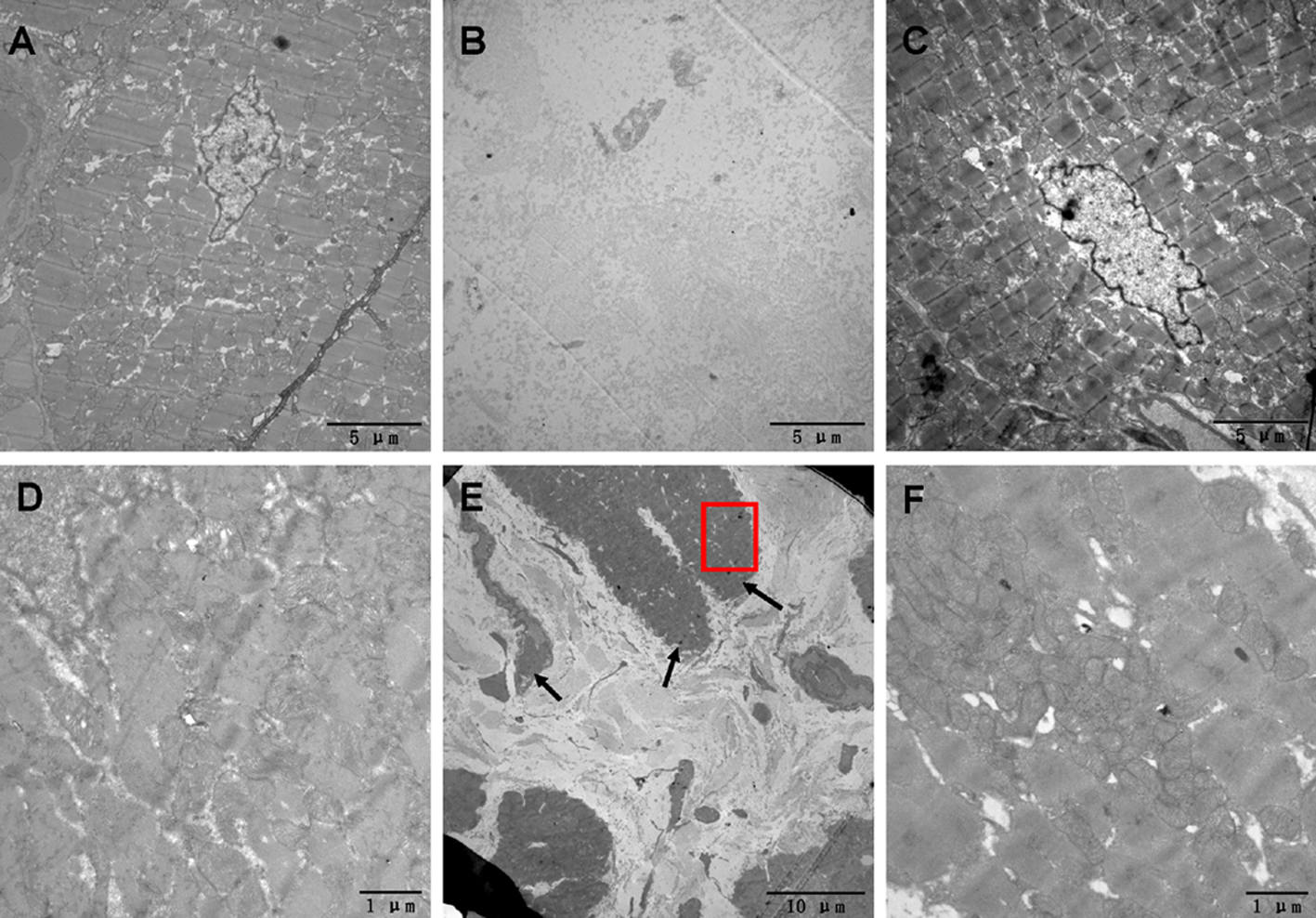

Supplement: Figure S4 — Ultrastructure of the myocardium in different groups. (A) Healthy myocardium from the Sham group. (B) Myocardium taken from the infarct zone of the PBS group showed a large damaged area. (C) Myocardium taken from the remote zone of the iPS group. (D) Myocardium taken from the border zone of the iPS group was only slightly damaged. (E) Myocardium taken from the infarct zone of the iPS group was mainly composed of fibroblasts, and newly formed myocardium also appeared (showed by red squared portion). (F) A highly magnification photograph of the squared portion of E. No tumor signs were seen. (TIF) [file pone.0066688.s004.tif]
