# Supplementary material for: Intramyocardial Injection of Pig Pluripotent Stem Cells Improves Left Ventricular Function and Perfusion: A Study in a Porcine Model of Acute Myocardial Infarction
Source: PLoS One. 2013 Jun 21;8(6):e66688. doi: 10.1371/journal.pone.0066688 (PMC3689724; doi:10.1371/journal.pone.0066688)
Supplement: Table S1 — The characteristics and hemodynamic data at the end of the 7th week after piPS cell transplantation. (DOC) [file pone.0066688.s005.doc]

**Supplemental Table S1. The characteristics and hemodynamic data at the end of the 7th week after piPS cell transplantation**.

|  | **Sham group** | **PBS group** | **iPS group** | ***p* values** |
| --- | --- | --- | --- | --- |
| Male* | 2 | 4 | 4 | 0.589 |
| BW at baseline (Kg) | 24.58±2.46 | 24.50±1.84 | 25.42±3.38 | 0.804 |
| BW on the 7th week (Kg) | 37.67±2.09 | 35.33±1.81 | 37.08±3.50 | 0.293 |
| LVESP (mmHg) | 106.67±10.80 | 94.77±16.50 | 103.47±16.48 | 0.380 |
| LVEDP (mmHg) † | 8.33±2.88 | 17.83±8.45 | 9.67±3.33 | 0.026 |
| Ao-SP (mmHg) † | 108.62±15.04 | 112.38±21.95 | 112.83±13.89 | 0.696 |
| Ao-DP (mmHg) | 83.33±5.65 | 85.39±18.62 | 81.00±14.873 | 0.867 |

Data are presented as mean ± SD. Only BW data was presented at baseline, and 7th week. * compared using Fisher's Exact Test. † compared using Kruskal-Wallis Test, all others were compared using ANOVA. BW= body weight; LVESP = left ventricular end-systolic pressure; LVEDP = left ventricular end-diastolic pressure; Ao-SP = aortic systolic pressure; Ao-DP = aortic diastolic pressure.
