# Supplementary material for: Intramyocardial Injection of Pig Pluripotent Stem Cells Improves Left Ventricular Function and Perfusion: A Study in a Porcine Model of Acute Myocardial Infarction
Source: PLoS One. 2013 Jun 21;8(6):e66688. doi: 10.1371/journal.pone.0066688 (PMC3689724; doi:10.1371/journal.pone.0066688)
Supplement: Table S2 — LV function parameters at the end of 1st and 6th week after piPS cell transplantation. (DOC) [file pone.0066688.s006.doc]

**Supplemental Table S2. LV function parameters at the end of 1st and 6th week after piPS cell transplantation.**

|  | **Sham group (n=6)** | **PBS group (n=6)** | **iPS group (n=6)** | ***p* values** |
| --- | --- | --- | --- | --- |
| **LVEDV (ml)** |  |  |  |  |
| 1st week | 71.17±14.77 | 70.17±22.61 | 76.17±9.43 | 0.799 |
| 6th week | 80.33±9.67 | 89.33±11.34 | 89.00±9.12 | 0.247 |
| **LVESV (ml)** |  |  |  |  |
| 1st week | 26.83±6.52 | 33.67±10.09 | 37.33±7.17 | 0.107 |
| 6th week | 29.50±4.76 | 44.17±8.59 | 38.83±7.57 | 0.010 |
| **SV (ml)** |  |  |  |  |
| 1st week | 44.33±9.00 | 36.50±12.85 | 38.83±3.66 | 0.352 |
| 6th week | 50.83±5.85 | 45.17±3.19 | 50.17±2.86 | 0.065 |
| **LVEF (%)** |  |  |  |  |
| 1st week | 62.47±3.46 | 51.72±3.09 | 51.25±3.94 | <0.001 |
| 6th week* | 63.37±3.26 | 50.93±3.91 | 56.68±4.44 | 0.002 |
| **MHR(bpm)** |  |  |  |  |
| 1st week | 95.17±17.69 | 106.33±29.76 | 114.33±11.43 | 0.313 |
| 6th week | 107.33±11.00 | 101.67±10.54 | 98.67±10.56 | 0.386 |
| **CO** |  |  |  |  |
| 1st week | 4.30±1.43 | 3.86±1.49 | 4.44±0.62 | 0.711 |
| 6th week | 5.46±0.81 | 4.60±0.60 | 4.95±0.58 | 0.120 |

Data are presented as mean ± SD. All of the data except LVEF on the 6th week exhibit homogeneity of variance, so the analysis of variance (ANOVA) test was performed to compare among groups. * compared using Kruskal-Wallis Test. LVEDV = left ventricular end-diastolic volume; LVESV = left ventricular end-systolic volume; SV = stroke volume; LVEF = left ventricular ejection fraction; MHR = mean heart rate; CO = cardiac output.
