# Supplementary material for: Intramyocardial Injection of Pig Pluripotent Stem Cells Improves Left Ventricular Function and Perfusion: A Study in a Porcine Model of Acute Myocardial Infarction
Source: PLoS One. 2013 Jun 21;8(6):e66688. doi: 10.1371/journal.pone.0066688 (PMC3689724; doi:10.1371/journal.pone.0066688)
Supplement: Text S1 — Supporting Materials and Methods. (DOC) [file pone.0066688.s007.doc]

**Supporting Information**

**TITLE:** **Intramyocardial injection of pig pluripotent stem cells improves left ventricular function and perfusion: a study in a porcine model of acute myocardial infarction**

**AUTHORS:**

Xiaorong Li**^†^**, Fengxiang Zhang**^†^**, Guixian Song, Weijuan Gu, Minglong Chen, Bing Yang, Dianfu Li, Daowu Wang, Kejiang Cao^*^

**Supporting Materials and Methods**

**Cell culture and preparation**

For our experiments, porcine iPS (piPS) cell lines, kindly provided by Professor Lei Xiao (Shanghai Institutes for Biological Sciences, Chinese Academy of Sciences, Shanghai, P. R. China), were generated from 10-week-old Danish Landrace pig primary ear ﬁbroblasts (PEFs) by drug-inducible expression of deﬁned factors including Oct4, Sox2, Klf4, c-Myc, Nanog and Lin28 . The cell culture methods and basic features of piPS cells have been described specifically by Wu et al . In brief, the undifferentiated piPS cells were routinely cultivated and propagated on irradiated CF-1 mouse embryonic fibroblasts (MEFs, SiDanSai, Shanghai, P. R. China) in DMEM/F12 supplemented with 20% knockout serum replacement, 1 mM L-glutamine, 0.1 mM beta-mercaptoethanol, 0.1 mM nonessential amino acid (all from Invitrogen, Carlsbad, California, USA), and 2 µg/ml doxycycline. MEFs were previously cultured in DMEM supplemented with 10% foetal bovine serum (FBS). MEF and piPS cells cultures were maintained at 37°C, 5% CO_2_ and 85% relative humidity. The piPS cell culture medium was changed every day. Every 3 to 4 days the piPS cells were passaged at a ratio of 1:3. Colonies were detached with collagenase IV (GIBCO-BRL) and plated again onto MEFs.

**AMI model creation and piPS cell transplantation**

All male or female domestic pigs (2-3 month of age) were obtained from Jiangsu Academy of Agricultural Sciences (Nanjing, P. R. China). An ear tag provided unique identification for each candidate pig, which was housed in the Laboratory Animal Center of Nanjing Medical University where lighting, room temperature, and humidity were comfortable. The specific method for AMI model creation has been previously described . Briefly, on the day of experimentation, the pigs were anesthetized initially with ketamine hydrochloride (20-25 mg/kg, intramuscularly, Fujian Gutian Medicine Co, Ltd, Gutian, P. R. China), diazepam (0.5 mg/kg intramuscularly, Tianjin Jinyaoanjisuan Co, Ltd, Tianjin, P. R. China) and atropine sulfate (0.4 mg/kg, intramuscularly, Shanghai Hefeng Medicine Co, Ltd, Shanghai, P. R. China) and then weighed. The anesthesia was then maintained with 3% pentobarbital sodium (Sigma-Aldrich, St. Louis, USA) and fentanyl (12.5 mg/kg/h, Yichang Renfu Medicine Co, Ltd, Yichang, P. R. China). When the animals were moved to the catheter room, limbs were bound and the pigs were placed onto the surgical table. Pigs were first given 8000 UI of heparin and the AMI was induced by occluding the distal third of left anterior descending coronary artery (LAD) just above the second diagonal branch for 90 minutes by inflation of the over-the-wire coronary balloon catheter.

AMI was conﬁrmed by continuous 12-lead Electrocardiography (ECG) which was used to monitor heart rate, rhythm, and showed typical persistent ST segment elevation during the procedure. Lidocaine or amiodarone was injected intravenously when pre-ventricular contractions (PVCs) or ventricular tachycardia (VT) occurred after the occlusion. Once ventricular fibrillation (VF) occurred, direct defibrillation (200 J) was quickly performed to convert the rhythm. AMI was also conﬁrmed by abnormal wall movement, which was evaluated by left ventricular (LV) angiography at baseline, and immediately after AMI.

Before transplantation, piPS cells were dissociated with 1 mg/ml type IV collagenase and cultured in the conditioned medium (CM) for 3 to 5 days. The piPS cell clones were collected and centrifuged at 1000 rpm, then re-suspended in PBS to obtain a concentration of 2×10^7^ cells in 2 ml. The cell suspension was aspirated into 1ml tuberculin syringes when cells were going to be injected. These piPS cells were labeled by a promoter-driven EGFP reporter to track the transplanted cells. On the day of transplantation, all pigs were sedated with ketamine hydrochloride and barbital sodium after an injection of 20 mg succinylcholine. Animals were then intubated with an endotracheal tube and mechanically ventilated with 100% oxygen to maintain PaCO_2_ between 35 and 45 mmHg by a volume-controlled ventilator. After the heart was exposed through midthoracotomy under sterile conditions, the pericardium was gently cut with a small incision and then fixed with chest wall. After intravenous injection of lidocaine hydrochloride (0.5 mg/kg), pigs in the iPS group received 2 × 10^7^ piPS cells suspended in 2 ml of sterile PBS solution that was injected directly in the center of the infarct zone (IZ) for 8 sites and along the border zone (BZ) for 12 sites (0.1 ml per injection, 1×10^6^ cells per site) (Figure 1C-D). The same volume of PBS was injected into the corresponding sites in the PBS group. Sham-operated animals, in which LAD was not occluded, were used as normal controls. Only the thoracic cavity was opened and the heart exposed, no PBS or piPS cells were injected. MI animals received cyclosporine (10 mg/kg per day) after cell or PBS delivery .

**Myocardial perfusion assessed by SPECT**

To determine myocardial perfusion, a serial resting ECG-gated ^99m^Tc-SPECT MIBI was performed before the experiment and at the end of the 1st and 6th week after cell transplantation, using 3-head gamma cameras equipped with low-energy high-resolution collimators (ECAM+, Siemens). These methods have been previously described . Briefly, after 8 hours of fasting, all pigs were sedated with intramuscular ketamine hydrochloride (20-25 mg/kg) and maintained with intravenous 3% sodium pentobarbital before examination as described in the model creation method. After anesthesia, 1 ml of 5 mCI ^99m^Tc-SPECT MIBI (^99m^Tc-MIBI) solution was injected into the ear vein. Forty-five minutes later, pigs were positioned laying on their right side with the heart in the examination bed to perform SPECT scan. According to the recommendation of the American College of Cardiology/American Heart Association, a 17-segment model based on the perfusion defect was used to score . The myocardial perfusion score was calculated using a ﬁve-point scoring system as previously described by Poornima et al . In the scoring system, 0 represents absent perfusion; 1 represents severe hypoperfusion; 2 represents moderate hypoperfusion; 3 represents mild hypoperfusion; and 4 represents normal perfusion. The total LV was divided into 17 segments, 7 of which were assigned to the LAD distribution area for assessment of the myocardium . The perfusion score for LV was graded from 0 to 28, indicating no perfusion (0) to normal perfusion (28). All SPECT images were analyzed and interpreted independently by two nuclear cardiologists, who were blinded to the study designation.

**Cardiac function assessed by dual source CT (DSCT) examination**

A previous study demonstrated that DSCT with ECG editing could provide results comparable to those of 2D-TTE for assessment of LV global function in patients even in case of severe arrhythmia . DSCT has established a role in the accurate detection of coronary artery disease and can accurately detected whether the myocardium was infracted . DSCT was performed to determine the coronary anatomy, myocardium infarction and cardiac function on the 1st and 6th week after cell delivery using a 64-slice DSCT scanner (Somatom Deﬁnition, Siemens Healthcare, Erlangen, Germany). The DSCT scan protocol and image reconstruction for patients described in our previous study was also used in the present study for the porcine AMI model examination. In brief, the pigs were positioned in the center of the scanner after another round of anesthesia, intubation and mechanical ventilation as described above. Next, 50-80 ml of contrast agent iopromide injection (Ultravist 370, Bayer Schering Pharma, Berlin, Germany, 2 ml/kg) at a concentration of 270 mg/mL was intravenously injected to the auricular vein, followed by flushing with 50 ml of saline. Both were delivered at a flow rate of 5 ml/s through a 22-GA BD Saf-T-Intima^TM^ using a dual-head power injector to conduct the contrast enhanced CT scan using dual-energy mode. Lidocaine or ß-blocker injections were given intravenously before examination if the heart rate (HR) was >130 beats per minutes or there were too many pre-ventricular contractions (PVCs). When the scan began, the ventilator was stopped to maintain an adequate time for the breath hold. The scanning was automatically triggered by a bolus tracking technique included in the scanner. The region of interest (ROI) was placed in the root of the aorta and image acquisition started 5 seconds after the CT attenuation reached the triggering threshold of 100 Hounsﬁeld Units (HU) at 100 kVp. The parameters for DSCT scan were as follows: 1) detector collimation width was 64×0.6 mm; 2) slice thickness was 0.6 mm; 3) rotation time was 330 seconds; 4) pitch was set at 0.2-0.5 according to the HR; 5) tube voltages were 120 kVp; and 6) effective tube currents were 400 mA, respectively.

At last, all images were copied and transferred to an external workstation MNS’ Navigant Software version 2.11 (Stereotaxis, Inc. St. Louis, MO), and analyzed in a blind fashion by two independent doctors. Each doctor had at least 3 years of experience in coronary CT angiography, and came from the Department of Diagnostic Radiology of the 1^st^ affiliated hospital of Nanjing Medical University.

To determine the LV function, end-diastolic phases (EDP, the phase with the largest LV cavity), and end-systolic phases (ESP, the phase with the smallest LV cavity) in each pig were automatically set by the software at 20 and 80% of the RR-interval. The tracing procedure for a smooth, concentric endocardial contour was automatically performed and adjusted manually if necessary (Fig. 3A-B). LV functional related parameters such as LV stroke volume (SV), cardiac output (CO), LV end-diastolic volume (LVEDV), LV end-systolic volume (LVESV), and LV ejection fraction (LVEF) were calculated using the software. SV is calculated as SV = EDV- ESV. The CO is calculated as CO = HR * SV. The LVEF is calculated as EF = (SV/EDV) * 100% .

**Hemodynamic data acquisition**

After the catheter was introduced to the LV on the 7th week after cell transplantation, hemodynamic data, including heart rate (HR), LV pressure (LVP), and aortic pressure (AOP) were acquired and processed using a specific analysis system.

**Gross pathology and histology examination**

Immediately after the hemodynamic examination, pigs were humanly sacrificed by intravenous administration of 20 ml of a 10% saturated potassium chloride (KCl) solution. To determined the infarct size and perform the histology examination, the chest was opened through the fourth intercostal space via a left thoracotomy. The whole heart was quickly excised and placed in cooled saline (4°C) for 30 minutes. The myocardium of the LV was then cut into 5-10 mm slices in the short axis. All slices were immersed in a 500 ml solution of 1% triphenyltetrazolium chloride (TTC, Sigma-Aldrich, St. Louis, USA) made fresh in PBS (pH 7.4). Subsequently, slices were bathed at 37°C for 30 minutes and then ﬁxed in 4% formalin solution. The slices were photographed under room light with a digital Canon camera. For each pig, the infarct size was measured as the sum of the volumes of all infarcts in all slices and was expressed as a percentage of the infarct area of the left ventricle. Thereby, it was calculated as: [infarct size=infarct area / (infarct area + non-infarct myocardial area) × 100%] . The infarct zone was demarcated and analyzed by planimetry using image analysis software (Image J, version 1.44, NIH, Bethesda, MD, USA. <http://rsbweb.nih.gov/ij/>). The remaining cardiac tissue was placed in 10% formalin for paraffin embedding and processed for immunohistochemistry staining (IHC).

After the death of the pigs, their hearts and other main organs such as the liver, pancreas, spleen, kidneys, and part of the lungs were taken and washed with cooled saline (4°C) for five minutes. Each organ was cut into several slices every 1 cm and photographed for examination of tumor formation. Part of the organs were cut into 1 × 1 × 0.3 cm^3^ cube for pathological examination. The tissue samples from the infarct heart were divided into three zones: the infracted zone (IZ), border zone (BZ) and remote zone (RZ) (Figure 1D). Subsequently, all cubed tissues were fixed with 4% buffered paraformaldehyde (PFA, pH 7.4) for 72 hours, followed by embedding in paraffin. These tissues were cut into 3-5 mm thick sections, which were mounted on positively charged glass slides (Beyotime Institute of Biotechnology, Nantong, P. R. China). After deparaffinization in xylene1 and rehydration *via* series of 100, 100, 95, 90, 80, and 70% ethanol solutions, they were stained with hematoxylin-eosin (Zhongshan Goadenbrige Biotechnology Inc., Beijing, P. R. China) to conﬁrm the infarction and morphology. In addition, tissue samples from the three zones of each heart were stained with Masson’s trichrome reagent kit (Maxim Bio Inc., Fuzhou, P. R. China) to determinate the collagen content according to previous reports .

**Analysis of transplanted cell differentiation and myocardial vascular density by immunofluorescence (IF) and IHC reactions**

To trace the transplanted cells, LV tissue samples collected from the IZ, BZ and RZ, specifically at the sites of cell or PBS injection, were in part frozen in liquid nitrogen immediately and then embedded in OCT medium when necessary. Serial sections were cut into 4 mm thickness to prepare for the double indirect IF. After 4% PFA (pH 7.4, 4 °C) ﬁxation, sections were incubated with the following primary antibodies: anti-a-sarcomeric actinin (1:400, Sigma-Aldrich, St. Louis, USA), monoclonal smooth muscle actinin (1:400, Sigma-Aldrich, St. Louis, USA), anti-α-smooth muscle cell actin (α-SMA) (1:500, Abcam, Hongkong, P. R. China) and GFP (1:100, Abcam, Hongkong, P. R. China). Overnight incubation at 4 °C was followed by was FITC or TRITC-conjugated secondary antibodies raised in goat (1:200, Sigma-Aldrich, St. Louis, USA) for 45 min at room temperature. All primary antibodies were diluted in an antibody dilution solution (Zhongshan Goadenbrige Biotechnology Inc., Beijing, P. R. China). Nuclei were counterstained with DAPI (1:1,000; Roche, Shanghai, P. R. China) for 15 min at room temperature. Subsequently, laser confocal microscopy ((LSM 510; Carl Jena, Germany) was used to take photos.

To assess vascular density, IHC reactions were performed according to an indirect immunoperoxidase method. The sections were deparafﬁnized, dehydrated, and washed in PBS. Following the antigen retrieval procedures, all the slides were incubated in 3% hydrogen peroxide (Zhongshan Goadenbrige Biotechnology Inc., Beijing, P. R. China) for 30 minutes at room temperatures to inhibit the intrinsic peroxidase activity. Next, the sections were blocked with 5% normal goat serum to eliminate nonspecific binding. The sections were then incubated with the mouse monoclonal SMA (Abcam, Hong Kong, P. R. China) antibody in humidity chambers overnight at 4°C. After the second day of incubation, the primary antibodies were recycled. All sides were washed with PBS–Tween-20 three times for 15 minutes. Next, all sections were subsequently incubated with the peroxidase-conjugated secondary antibody (goat anti-mouse IgG (H+L), 1:100, Jackson Immunoresearch Laboratories) at a dilution of 1:100 for 45 minutes at room temperature. This was followed by staining using diaminobenzidine chromogen (DAB) at room temperature for 5-10 minutes. Finally, the nuclei were counterstained with hematoxylin for 10 minutes, dehydrated, cleared in xylene, and covered with a coverslip. The capillary density was determined by calculating three fields/section taken in a blind fashion in the IZ at 200×magnification. A total of three sections per animal and three animals per group were assessed. The analysis was performed with the aid of Image J software as previously described. The amount of capillaries was expressed as per 200×field.

**Transmission electron microscopy examination (EM) to detect ultrastructural alterations**

Transmission electron microscopy was performed as previously described . Briefly, animals were kindly sacrificed by an injection of KCL. After opening the pericardium, the myocardial samples were taken from three zones and quickly rinsed in 0.9% sodium chloride and prepared for electron microscopy. Small (1 mm^3^ cubes) transmural ventricular tissue (n=3 per group) were fixed in buffered 5% glutaraldehyde in 0.1 M cacodylate buffer (pH 7.4) at 4 °C for 24 h to 72 h. Samples were then postfixed in 1% OsO_4_ and followed by dehydration *via* a series of 50, 70, 90 and 100% ethanol solutions. They were then infiltrated by propylene oxide and embedded in Epon 812. Thin sections (~60 nm) were cut with an ultra-microtome, placed on a grid and after staining with lead acetate, viewed in a transmission electron microscope (JEM1010; JEOL Ltd, Tokyo, Japan) at 80 kV with magnification ×6000，20,000 and 40,000. In total, two samples per animal and three animals per group were selected to assess the ultrastructural alterations. All micrographs were examined by an individual from the Department of Electron Microscope of Nanjing Medical University and confirmed by another individual. Both individuals were blind to this experiment.

**References**

1. Wu Z, Chen J, Ren J, Bao L, Liao J, et al. (2009) Generation of pig induced pluripotent stem cells with a drug-inducible system. J Mol Cell Biol 1: 46-54.

2. Ellison GM, Torella D, Dellegrottaglie S, Perez-Martinez C, Perez de Prado A, et al. (2011) Endogenous cardiac stem cell activation by insulin-like growth factor-1/hepatocyte growth factor intracoronary injection fosters survival and regeneration of the infarcted pig heart. J Am Coll Cardiol 58: 977-986.

3. Zeng L, Hu Q, Wang X, Mansoor A, Lee J, et al. (2007) Bioenergetic and functional consequences of bone marrow-derived multipotent progenitor cell transplantation in hearts with postinfarction left ventricular remodeling. Circulation 115: 1866-1875.

4. Tao Z, Chen B, Tan X, Zhao Y, Wang L, et al. (2011) Coexpression of VEGF and angiopoietin-1 promotes angiogenesis and cardiomyocyte proliferation reduces apoptosis in porcine myocardial infarction (MI) heart. Proc Natl Acad Sci U S A 108: 2064-2069.

5. Kim BO, Tian H, Prasongsukarn K, Wu J, Angoulvant D, et al. (2005) Cell transplantation improves ventricular function after a myocardial infarction: a preclinical study of human unrestricted somatic stem cells in a porcine model. Circulation 112: I96-104.

6. Valina C, Pinkernell K, Song YH, Bai X, Sadat S, et al. (2007) Intracoronary administration of autologous adipose tissue-derived stem cells improves left ventricular function, perfusion, and remodelling after acute myocardial infarction. Eur Heart J 28: 2667-2677.

7. Cerqueira MD, Weissman NJ, Dilsizian V, Jacobs AK, Kaul S, et al. (2002) Standardized myocardial segmentation and nomenclature for tomographic imaging of the heart: a statement for healthcare professionals from the Cardiac Imaging Committee of the Council on Clinical Cardiology of the American Heart Association. Circulation 105: 539-542.

8. Poornima IG, Miller TD, Christian TF, Hodge DO, Bailey KR, et al. (2004) Utility of myocardial perfusion imaging in patients with low-risk treadmill scores. J Am Coll Cardiol 43: 194-199.

9. Kim SS, Ko SM, Song MG, Kim JS (2010) Assessment of global function of left ventricle with dual-source CT in patients with severe arrhythmia: a comparison with the use of two-dimensional transthoracic echocardiography. Int J Cardiovasc Imaging 26: 213-221.

10. Scheffel H, Alkadhi H, Plass A, Vachenauer R, Desbiolles L, et al. (2006) Accuracy of dual-source CT coronary angiography: First experience in a high pre-test probability population without heart rate control. Eur Radiol 16: 2739-2747.

11. Rubinshtein R, Miller TD, Williamson EE, Kirsch J, Gibbons RJ, et al. (2009) Detection of myocardial infarction by dual-source coronary computed tomography angiography using quantitated myocardial scintigraphy as the reference standard. Heart 95: 1419-1422.

12. Li C, Tang L, Yang Z, Cao K (2011) Integration of dual source computed tomography With magnetic navigation system for percutaneous coronary intervention: A feasibility study. Catheter Cardiovasc Interv 78: 1108-1115.

13. Groen JM, van der Vleuten PA, Greuter MJ, Zijlstra F, Oudkerk M (2009) Comparison of MRI, 64-slice MDCT and DSCT in assessing functional cardiac parameters of a moving heart phantom. Eur Radiol 19: 577-583.

14. Merrill GF, Rork TH, Spiler NM, Golfetti R (2004) Acetaminophen and myocardial infarction in dogs. Am J Physiol Heart Circ Physiol 287: H1913-1920.

15. Oron U, Yaakobi T, Oron A, Mordechovitz D, Shofti R, et al. (2001) Low-energy laser irradiation reduces formation of scar tissue after myocardial infarction in rats and dogs. Circulation 103: 296-301.

16. Koch KC, Schaefer WM, Liehn EA, Rammos C, Mueller D, et al. (2006) Effect of catheter-based transendocardial delivery of stromal cell-derived factor 1alpha on left ventricular function and perfusion in a porcine model of myocardial infarction. Basic Res Cardiol 101: 69-77.

17. Ahmed RP, Ashraf M, Buccini S, Shujia J, Haider H (2011) Cardiac tumorgenic potential of induced pluripotent stem cells in an immunocompetent host with myocardial infarction. Regen Med 6: 171-178.
